# 中国人群肺鳞癌患者免疫治疗疗效及不良反应观察

**DOI:** 10.3779/j.issn.1009-3419.2022.101.36

**Published:** 2022-07-20

**Authors:** 江泳 于, 晓楠 武, 俊玲 马, 晞 陈, 琳 李

**Affiliations:** 100730 北京，北京医院肿瘤科，国家老年医学中心，中国医学科学院老年医学研究院 Department of Oncology, Beijing Hospital, National Center of Gerontology; Institute of Geriatric Medicine, Chinese Academy of Medical Science, Beijing 100730, China

**Keywords:** 肺肿瘤, 免疫治疗, 疗效, 不良反应, Lung neoplasms, Immunology therapy, Efficacy, Adverse effects

## Abstract

**背景与目的:**

免疫检查点抑制剂（immune checkpoint inhibitors, ICIs）改善了部分肺鳞状细胞癌（肺鳞癌）患者的生存，目前仍缺乏高龄患者免疫治疗的数据。本研究探讨真实世界中不同年龄肺鳞癌患者免疫治疗的疗效和不良反应。

**方法:**

回顾性分析2018年1月-2022年1月就诊于北京医院经病理明确诊断的肺鳞癌患者185例，124例接受内科一线治疗，其中化疗组57例，免疫联合化疗组（联合组）46例，单药免疫组13例，靶向治疗组8例。按年龄分为高龄组（年龄≥70岁）73例和低龄组（年龄 < 70岁）112例。比较高龄组与低龄组患者以及不同治疗方式疗效和不良反应。评价程序性死亡配体1（programmed cell death ligand 1, PD-L1）和肿瘤突变负荷（tumor mutational burden, TMB）表达作为疗效、预后标志物的价值。采用实体瘤疗效评价标准（Response Evaluation Criteria in Solid Tumors version 1.1, RECIST 1.1）评价近期疗效，常见不良反应分级评价标准（Common Terminology Criteria for Adverse Events version 4.03, CTCAE 4.03）评价免疫相关不良反应。*Kaplan*-*Meier*法绘制生存曲线，并进行*Log*-*ran*k检验。*Cox*风险比例回归模型分析影响预后的因素。

**结果:**

高龄组患者一线联合组总有效率（overall response rate, ORR）为69.2%（9/13），显著高于化疗组的25%（4/16）（χ^2^=5.673, *P* < 0.05）。低龄组ORR为53.8%（14/26），高于化疗组的27.3%（9/33），差异不显著（χ^2^=4.317, *P* > 0.05）。高龄组单药免疫、联合组及化疗组的中位无进展生存时间（median progression-free survival, mPFS）和中位总生存时间（median overall survival, mOS）均与低龄组无差异（均*P* > 0.05）。高龄组一线联合组与免疫单药组mPFS均较化疗组延长，但差异均未达到统计学意义（均*P* > 0.05）。联合组及免疫单药组mOS与化疗组均无差异（均*P* > 0.05）。低龄联合组较化疗组显著延长mPFS（12.30个月 *vs* 6.17个月，*P* < 0.01）。mOS较化疗组明显延长（39.03个月 *vs* 14.4个月，*P* > 0.05）。高龄组PD-L1表达阳性率显著高于低龄组[82.6% (19/23) *vs* 54.3% (19/35), χ^2^=4.928, *P* < 0.05]。高龄组与低龄组TMB表达无差异（56.8% *vs* 52.0%, *P* > 0.05）。高龄组与低龄组PD-L1与TMB表达均与患者mPFS和mOS无关。高TMB表达患者中，联合组mPFS较化疗组显著延长（8.6个月 *vs* 3.5个月，*P* < 0.05），但mOS无差异（*P* > 0.05）。低TMB患者中，化疗组mPFS优于联合组，但未达到统计学差异（*P* > 0.05）。*Cox*风险比例回归模型提示临床分期是进展期肺鳞癌患者独立预后因子。总体免疫相关不良反应发生率为58.0%（*n*=51），3级及以上发生率25.0%（*n*=22）。最常见的3级及以上不良反应有皮疹、免疫相关性肺炎和乏力。高龄组与低龄组不良反应发生率无差异。

**结论:**

一线免疫联合化疗较化疗提高肺鳞癌患者ORR、mPFS和mOS。高龄组肺鳞癌患者免疫单药或联合化疗疗效与低龄组相似，总体不良反应可控。

## Abstract (en)

**Background and objective:**

Immune checkpoint inhibitors (ICIs) improved survival of partial patients with lung squamous cell carcinoma (LUSC). However, it was still insufficient of data in older patients. This study aimed to investigate the efficacy and toxicity of immunotherapy in patients with LUSC in Chinese population of real world.

**Methods:**

A total of 185 LUSC patients underwent pathological diagnosis were involved from January 2018 to January 2022. Patients were divided into elderly group (age ≥70 years) and younger group (age < 70 years). The efficacy of mono-immunotherapy or combined with chemotherapy to chemotherapy in first-line treatment was compared. The expression of programmed cell death ligand 1 (PD-L1) and tumor mutational burden (TMB) were evaluated. Response Evaluation Criteria in Solid Tumors (RECIST) version 1.1 was used to evaluate the efficacy, and Common Terminology Criteria for Adverse Events (CTCAE) version 4.03 was used to evaluate immune-related adverse. *Kaplan*-*Meier* and *Log*-*rank* test was performed. *Cox* regression was used in prognostic analysis.

**Results:**

Combined therapy acquired significantly higher overall response rate (ORR) compared with chemotherapy alone in elderly group (*P* < 0.05), and also in younger group, despite the difference was not significant (*P* > 0.05). The median progression-free survival (mPFS) and median overall survival (mOS) in elderly group were similar with younger group (*P* > 0.05). Both combined group and immunology alone demonstrated prolonged mPFS in first-line compared with chemotherapy in elderly group. And combined group demonstrated significantly prolonged mPFS compared with chemotherapy in younger group (*P* < 0.01). There was no difference of mOS between different regimes in two groups. Elderly LUSC patients had higher PD-L1 positive rate (≥1%) and similar TMB compared with younger group. There was no relationship between mPFS and mOS with the expression of PD-L1 and TMB. Immunology combined with chemotherapy demonstrated better mPFS compared to chemotherapy in first-line therapy with TMB-High (*P* < 0.05), and inferior mPFS with TMB-Low despite the difference was not significant (*P* > 0.05). *Cox* regression model demonstrated that clinical stage was an independent predictor and prognostic factor. The incidence of immune-related adverse was 58.0% (51/88) and grade 3 or above 25.0% (22/88). The most common grade 3 adverse events were rash, immune-associated pneumonia, and fatigue.

**Conclusion:**

Immunology combined with chemotherapy increased ORR, mPFS and mOS of Chinese patients with LUSC in first-line therapy compared with chemotherapy. There was no difference of efficacy and adverse effects rate between elderly group and younger group. The adverse effects of immunology in elderly patients with LUSC were controllable.

肺癌是我国发病率和死亡率最高的恶性肿瘤。《2020年全球癌症统计数据》报告中，肺癌在新发癌症患者中的发病率为11.4%，高居第二位；死亡率占比为18%，是全球死亡率最高的癌症类型^[1]^。中国癌症标化死亡率为122.2/10万，高于世界平均水平的102.4/10万。肺癌按组织学分为非小细胞肺癌（non-small cell lung cancer, NSCLC）和小细胞肺癌两大类，NSCLC占全部肺癌患者的85%。早期肺癌多无明显症状，多数患者就诊时已属晚期，整体5年生存率只有15%。传统含铂双药化疗有效率低，缓解时间短，生存获益有限^[2]^。近年来，以抗程序性死亡受体/配体1（programmed cell death protein 1/programmed cell death ligand 1, PD-1/PD-L1）和抗细胞毒T淋巴细胞抗原4（cytotoxic T-lymphocyte-associated protein 4, CTLA-4）为代表的免疫检查点抑制剂（immune checkpoint inhibitors, ICIs）在肺癌中发展迅速。多项临床研究^[3, 4]^结果表明，ICIs改善了部分NSCLC患者的疗效和预后，甚至可达到长期生存。

肺鳞状细胞癌（肺鳞癌）是最常见的肺癌组织学亚型之一，约占NSCLC患者的30%^[5]^。肺鳞癌早期缺乏明显症状，大多数患者确诊时已属晚期，丧失手术机会。与肺腺癌不同，只有极少数肺鳞癌患者存在表皮生长因子受体突变或间变性淋巴瘤激酶基因重排等驱动基因，因此缺乏靶向药物治疗机会。近年来，多项临床研究结果^[6-8]^均表明，相对于传统化疗，ICIs单药或联合化疗的方案可明显改善晚期肺鳞癌患者治疗有效率和生存时间，甚至引起肺鳞癌患者治疗模式的改变。然而，另两项Ⅲ期研究^[9, 10]^分别探讨Nivolumab和Durvalumab在晚期NSCLC一线治疗的作用，虽然免疫治疗组总生存有获益趋势，但均未达到预设的统计学差异。另外，临床上仅15%-30%的晚期NSCLC患者可以从免疫治疗中获得持续缓解和长期生存。临床上如何有效筛选ICIs获益人群成为重要课题。

肺鳞癌多见于老年吸烟男性，40%的患者确诊时年龄超过70岁^[11]^。老年患者多伴有慢性合并症，脏器储备功能下降，化疗药物耐受性差^[12]^，具有独特的药代动力学和临床生物学特点。目前，多数ICIs相关临床研究中老年患者的入组人数有限，甚至排除老年患者。只有个别回顾性研究^[13]^或聚类分析探讨ICIs在老年肺鳞癌患者中的疗效和安全性^[14]^。本研究回顾性分析真实世界肺鳞癌患者免疫治疗疗效及不良反应情况，探讨高龄（≥70岁）肺鳞癌患者ICIs治疗疗效与安全性以及PD-L1、肿瘤突变负荷（tumor mutational burden, TMB）表达与疗效和预后的相关性。

## 1 资料与方法

### 1.1 临床资料

回顾性分析2018年1月-2022年1月就诊于北京医院经穿刺或手术病理明确诊断的肺鳞癌患者185例，中位年龄67岁（37岁-92岁）。男性171例（92.4%），女性14例（7.6%）。美国东部肿瘤协作组体力状况评分（Eastern Cooperative Oncology Group, Performance Status, ECOG-PS）：0分-1分179例，2分6例；82例（44.3%）患者并存冠心病、慢性阻塞性肺疾病、脑梗死、心律失常、其他肿瘤或肾功能不全等；147例（79.5%）患者有吸烟史；肿瘤原发灶-淋巴结-转移（tumor-node-metastasis, TNM）分期：Ⅰ期11例（5.9%），Ⅱ期20例（10.8%），Ⅲ期72例（38.9%），Ⅳ期82例（44.3%）；55例（29.7%）患者内脏转移；81例（43.8%）患者曾接受外科手术或介入消融等治疗。详见表 1。本研究为观察性非介入研究，经北京医院伦理委员会批准，入选者均签署知情同意书。

**表 1:** 185例肺鳞癌患者一般特征分析 Basic characteristics of 185 patients with lung squamous cell carcinoma

Characteristics	Data [*n* (%)]
Age (yr)	
Median age	67
Age range	37-92
Gender	
Male	171 (92.4)
Female	14 (7.6)
Smoking history	
No	38 (20.5)
Yes	147 (79.5)
ECOG-PS score	
0	33 (17.8)
1	146 (78.9)
2	6 (3.2)
Clinical stage	
Ⅰ	11 (5.9)
Ⅱ	20 (10.8)
Ⅲ	72 (38.9)
Ⅳ	82 (44.3)
Severe complications	
No	103 (55.7)
Yes	82 (44.3)
Operative treatment	
Surgery	71 (38.4)
Interventional	10 (5.4)
Radiotherapy	
No	131 (70.8)
Yes	54 (29.2)
PD-L1 expression (*n*=58)	
< 1%	20 (34.5)
1%-49%	25 (43.1)
≥50%	13 (22.4)
TMB (muts/Mb) (*n*=87)	
TMB-L (< 9)	40 (46.0)
TMB-H (≥9)	47 (54.0)
ECOG-PS: Eastern Cooperative Oncology Group-performance status; PD-L1: programmed cell death ligand-1; TMB-L: tumor mutation burden-low; TMB-H: tumor mutation burden-high	

### 1.2 方法

#### 1.2.1 临床分组

185例患者中124例接受内科一线治疗，其中化疗组57例，免疫联合化疗组（联合组）46例，单药免疫组13例，靶向治疗组8例。按年龄分为高龄组（年龄≥70岁）73例和低龄组（年龄 < 70岁）112例。

#### 1.2.2 治疗方案

① 化疗方案：白蛋白紫杉醇（石药集团欧意药业）130 mg/m^2^，第1、8天，21 d为1个周期；多西他赛（齐鲁制药）75 mg/m^2^或60 mg/m^2^，第1天，21 d为1个周期；紫杉醇脂质体（南京绿叶思科药业）135 mg/m^2^-175 mg/m^2^，第1天，21 d为1个周期；吉西他滨（江苏豪森药业）1, 000 mg/m^2^-1, 250 mg/m^2^，第1、8天，21 d为1个周期；长春瑞滨（法国皮尔法伯制药）25 mg/m^2^，第1、8天，21 d为1个周期；卡铂（齐鲁海南制药）按血药浓度时间曲线下面积（area under curve, AUC）为5-6，第1天，21 d为1个周期；②免疫治疗方案：纳武利尤单抗（美国百时美施贵宝公司) 3 mg/kg，第1天，21 d为1个周期；帕博利珠单抗（美国默沙东公司）200 mg，第1天，21 d为1个周期；阿替利珠单抗（美国罗氏公司）1, 200 mg，第1天，21 d为1个周期；信迪利单抗（信达生物制药）200 mg，第1天，21 d为1个周期；卡瑞丽珠单抗（江苏恒瑞医药）240 mg，第1天，21 d为1个周期；特瑞普利单抗（苏州众合生物医药）200 mg，第1天，21 d为1个周期；替雷丽珠单抗（广州百济神州生物制药）200 mg，第1天，21 d为1个周期。化疗组接受单药或含铂双药化疗，联合组在化疗基础上联合一种免疫治疗方案，单药免疫组接受一种免疫治疗药物。如果出现美国国立癌症研究所常见不良反应分级评价标准（National Cancer Institute-Common Toxicity Criteria for Adverse Events, NCI-CTCAE）4.03版本规定的3级-4级化疗不良反应，则下次化疗药物剂量调整至上一个周期剂量的75%。

### 1.3 观察指标

#### 1.3.1 检查随访

所有患者基线均评估头部核磁共振/计算机断层扫描（computed tomography, CT），胸、腹、盆腔增强CT或正电子发射计算机断层显像（positron emission computed tomography, PET-CT），血常规，肝肾功，电解质，心电图及骨扫描等检查。治疗期间治疗反应评估基于每2个月进行CT扫描，每6-12个月行骨扫描检查。

#### 1.3.2 疗效判定

临床疗效：采用NCI实体瘤疗效评价标准（Response Evaluation Criteria in Solid Tumors 1.1, RECIST 1.1）^[15]^，分为：①完全缓解（completed response, CR）：所有目标病灶消失；②部分缓解（partial response, PR）：目标病灶最长径之和与基线状态比较减少30%及以上；③疾病稳定（stable disease, SD）：介于部分缓解和疾病进展之间；④疾病进展（progressive disease, PD）：目标病灶最长径之和较治疗开始后记录的最小目标病灶最长径之和增加20%，或出现1个或多个新病灶。

总有效率（overall response rate, ORR）=（CR+PR）例数/总例数×100%，疾病控制率（disease control rate, DCR）=（CR+PR+SD）例数/总例数×100%。无进展生存时间（progression-free survival, PFS）：从治疗开始到治疗失败（疾病进展、死亡或出现不可耐受毒性）或最后一次随访的时间。总生存时间（overall survival, OS）：治疗开始直到任何原因的死亡或最后一次随访的时间。

#### 1.3.3 不良反应分级

采用NCI-CTCAE 4.03版本，分为1级-5级。

#### 1.3.4 PD-L1与TMB评价

采用DAKO 22C3 pharmDx免疫组化（immunohistochemistry, IHC）法评估PD-L1表达水平。可检测样本需满足100个肿瘤细胞以上的标准。结果根据细胞膜染色的程度和强度量化为肿瘤比例评分（tumor proportion score, TPS）。PD-L1表达阳性（TPS≥1%），PD-L1表达阴性（TPS < 1%）。TMB评价通过北京吉因加科技有限公司1021基因Panel，计算为非同义单核苷酸变异（single nucleotide variation, SNV）数量除以Panel覆盖基因组区域的长度（1.09 Mb）。高突变负荷（tumor mutational burden-high, TMB-H）：TMB≥9 mut/Mb，低突变负荷（tumor mutational burden-low, TMB-L）：TMB < 9 mut/Mb。

### 1.4 统计学方法

采用SPSS 19.0统计软件（IBM公司，Armonk，NY，USA），计数资料以例（%）表示，采用*Wilcoxon*秩和检验或χ^2^检验比较临床和病理参数的组间差异，临床疗效差异及药物不良反应比较采用χ^2^检验。采用*Kaplan*-*Meier*法绘制生存曲线，并进行*Log*-*rank*检验。多因素分析采用*Cox*风险比例回归模型。双侧*P* < 0.05为差异有统计学意义。

## 2 结果

### 2.1 临床不可手术或进展期肺鳞癌患者总体疗效与生存分析

临床不可手术或进展期肺鳞癌患者的中位总生存时间（median OS, mOS）为22.83个月。一线治疗可评价疗效的101例患者中，ORR为38.6%（*n*=39），DCR为76.2%（*n*=77）。患者一线治疗中位无进展生存时间（median PFS, mPFS）为7.13个月，mOS为15.73个月。

### 2.2 不同年龄组肺鳞癌患者一线治疗疗效分析

高龄组患者一线治疗ORR为38.9%（14/36），DCR为66.7%（24/36）。低龄组一线治疗ORR为38.5%（25/65），DCR为81.5%（53/65）。两组ORR与DCR均无统计学差异（χ^2^=0.002和χ^2^=2.829，均*P* > 0.05）。

高龄组患者一线联合治疗组ORR为69.2%（9/13），DCR为69.2%（9/13），化疗组ORR为25%（4/16），DCR为75%（12/16）。高龄联合组ORR显著高于化疗组（χ^2^=5.673, *P* < 0.05），DCR无明显差异（χ^2^=0.120, *P* > 0.05）。低龄组患者一线联合组ORR为53.8%（14/26），DCR为92.3%（24/26），化疗组ORR为27.3%（9/33），DCR为75.8%（25/33）。低龄联合组ORR和DCR高于化疗组，但均未达到统计学差异（χ^2^=4.317和χ^2^=2.830，均*P* > 0.05）。高龄组与低龄组一线联合治疗的ORR和DCR差异均无统计学意义（χ^2^=0.848和χ^2^=3.545，均*P* > 0.05）。

### 2.3 肺鳞癌患者一线治疗生存分析

#### 2.3.1 不同年龄组一线治疗生存分析

高龄组免疫单药治疗mPFS较低龄组延长（9.13个月 *vs* 2.0个月，*P* > 0.05）。高龄联合组mPFS低于低龄组（8.77个月 *vs* 12.30个月，*P* > 0.05）。高龄组接受化疗患者mPFS低于低龄组（3.73个月 *vs* 6.17个月，*P* > 0.05）。

高龄组免疫单药治疗mOS与低龄组相似（9.97个月 *vs* 7.0个月，*P* > 0.05）。两组接受联合治疗患者mOS也没有明显差异（未达到 *vs* 39.03个月，*P* > 0.05）。高龄组接受化疗患者mOS较低龄组延长（25.67个月 *vs* 14.40个月，*P* > 0.05）。

#### 2.3.2 一线治疗不同治疗方式生存比较

如图 1A、图 1B所示，高龄组患者中，一线联合组与免疫单药组mPFS较化疗组延长，分别为8.77个月 *vs* 3.73个月，9.13个月 *vs* 3.73个月，差异均未达到统计学意义（*P*均 > 0.05）。低龄组患者中，一线联合组mPFS较化疗组明显延长（12.30个月 *vs* 6.17个月，*P* < 0.01）。免疫单药组与化疗组比较，mPFS为2.0个月 *vs* 6.17个月，差异无统计学意义（*P* > 0.05）。联合组mPFS较免疫单药组明显延长（12.30个月 *vs* 2.0个月），但差异无统计学意义（*P* > 0.05）。

**图 1:**
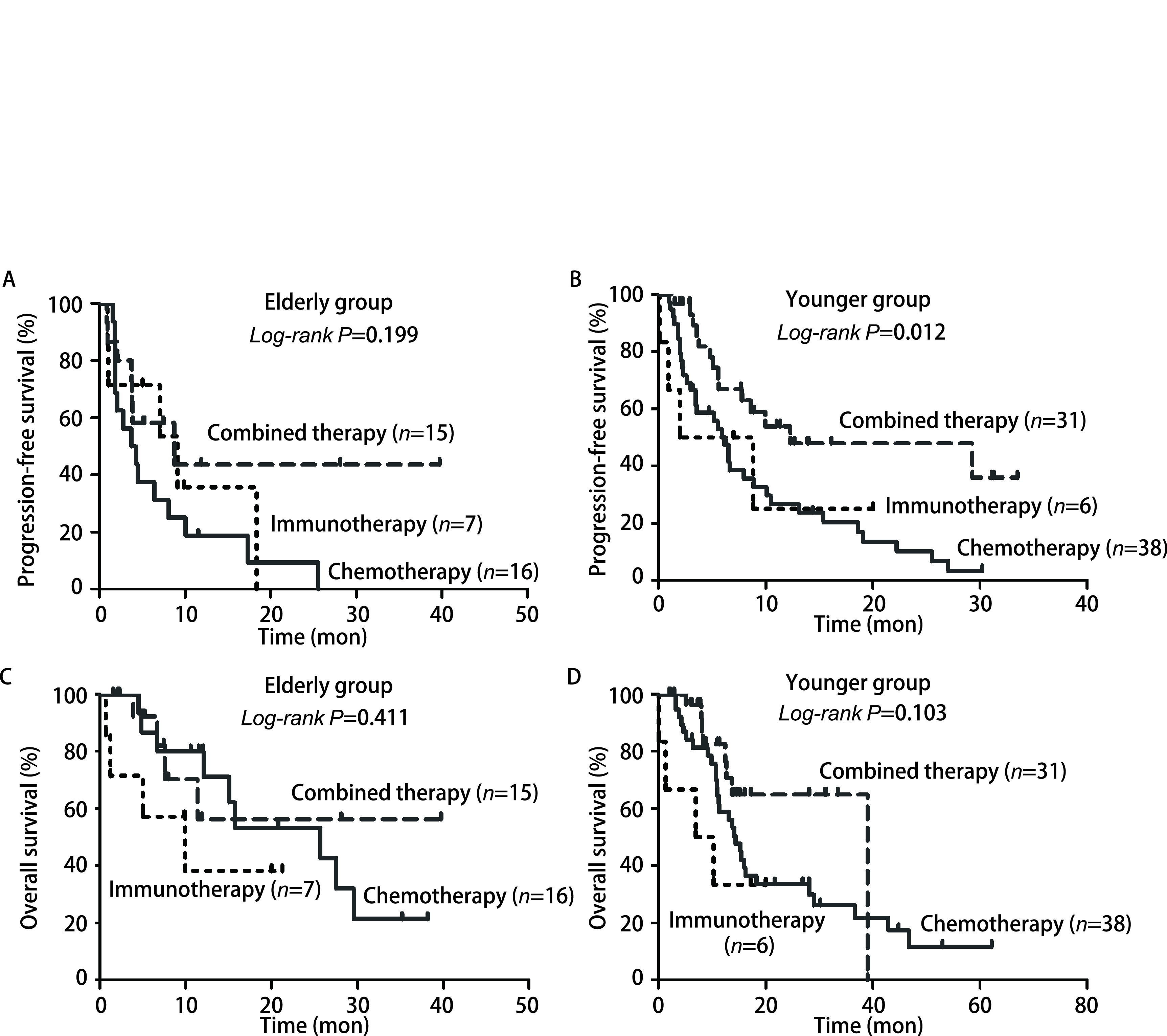
不同年龄组肺鳞癌患者生存分析。A：高龄组（≥70岁）患者一线治疗不同治疗方式的PFS比较；B：低龄组（< 70岁）患者一线治疗不同治疗方式的PFS比较；C：高龄组（≥70岁）患者一线治疗不同治疗方式的OS比较；D：低龄组（< 70岁）患者一线治疗不同治疗方式的OS比较。 Survival analysis of lung squamous cell carcinoma in different age groups. A: Comparison of first-line PFS between different regimes in elderly patients (≥70 years); B: Comparison of first-line PFS in younger patients (< 70 years); C: Comparison of first-line OS in elderly patients (≥70 years); D: Comparison of first-line OS in younger patients (< 70 years). PFS: progression-free survival; OS: overall survival.

如图 1C、图 1D所示，高龄组患者中，一线治疗联合组mOS与化疗组无明显差异（未达到 *vs* 25.67个月，*P* > 0.05）。联合组与化疗组mOS均较免疫单药组明显延长，分别为未达到 *vs* 9.97个月，25.67个月 *vs* 9.97个月，但差异无统计学意义（均*P* > 0.05）。低龄组患者中，一线治疗联合组mOS较化疗组明显延长（39.03个月 *vs* 14.4个月），但差异无统计学意义（*P* > 0.05）。联合组mOS较免疫单药组显著延长（39.03个月 *vs* 7.0个月，*P* < 0.05）。化疗组mOS较免疫单药组延长（14.40个月 *vs* 7.0个月），但差异无统计学意义（*P* > 0.05）。

### 2.4 不同年龄组肺鳞癌患者PD-L1与TMB表达情况

高龄组PD-L1表达阳性（≥1%）率显著高于低龄组[82.6% (19/23) *vs* 54.3% (19/35), χ^2^=4.928, *P* < 0.05]。高龄组PD-L1表达50.0%以上阳性率高于低龄组[30.4% (7/23) *vs* 17.1% (6/35)]，差异无统计学意义（χ^2^=1.410, *P* > 0.05）。高龄组与低龄组TMB表达无差异（*P* > 0.05），TMB-H患者分别为56.8%（21/37）*vs* 52.0%（26/50）。

### 2.5 肺鳞癌患者PD-L1与TMB表达与生存相关性

高龄组PD-L1表达阴性（< 1%）与阳性（≥1%）患者的mOS无差异（未达到 *vs* 23.03个月，*P* > 0.05）。低龄组PD-L1阴性mOS优于PD-L1阳性患者（31.53个月 *vs* 15.10个月），但差异无统计学意义（*P* > 0.05）。高龄组TMB-H较TMB-L患者延长mOS（41.63个月 *vs* 26.50个月），差异无统计学意义（*P* > 0.05）。低龄组TMB-H与TMB-L患者mOS无差异（44.13个月 *vs* 37.83个月，*P* > 0.05）。

PD-L1阳性患者中，联合组与化疗组mPFS和mOS差异均无统计学意义，分别为5.56个月 *vs* 4.47个月，未达到 *vs* 17.4个月（均*P* > 0.05）。TMB-H患者中，联合组mPFS较化疗组延长（8.6个月 *vs* 3.5个月，*P* < 0.05），但mOS无统计学差异（未达到 *vs* 16.03个月，*P* > 0.05）。TMB-L患者中，联合组mPFS低于化疗组（5.56个月 *vs* 13.13个月，*P* > 0.05），两组mOS无差异（未达到 *vs* 36.63个月，*P* > 0.05）。

### 2.6 影响疗效、预后的单因素与多因素分析（表 2）

**表 2:** 不可手术或进展期肺鳞癌患者生存相关单因素及多因素分析 Univariate and multivariate analysis of survival in patients with inoperable or advanced lung squamous cell carcinoma

Variables	First-line PFS				Overall survival		
	Univariate (*Log*-*rank*) *P*	Multivariate (*Cox* regression analysis)			Univariate (*Log*-*rank*) *P*	Multivariate (*Cox* regression analysis)	
		HR (95%CI)	*P*			HR (95%CI)	*P*
ECOG-PS (2 *vs* 0-1)	0.740	0.801 (0.103-6.222)	0.832		0.190	3.427 (0.390-30.151)	0.267
Age (≥70 *vs* < 70)	0.196	0.530 (0.267-1.050)	0.069		0.483	0.473 (0.186-1.205)	0.117
Clinical stage (Ⅳ *vs* Ⅲ)	0.003	2.579 (1.322-5.033)	0.005		0.005	4.596 (1.651-12.792)	0.003
TMB (≥9 mut/Mb *vs* < 9 mut/Mb)	0.487	0.686 (0.362-1.300)	0.248		0.480	0.520 (0.218-1.240)	0.140
PD-L1 expression (≥1% *vs* < 1%)	0.319	-	-		0.824	-	-
PD-L1: programmed cell death ligand-1; TMB: tumor mutational burden; HR: hazard ratio; -: irrelevant factor.							

单因素分析结果显示，影响患者OS的因素为临床分期，进一步*Cox*比例风险模型多因素分析结果显示仍只有临床分期是独立预后因素。单因素和多因素分析结果均提示，只有临床分期是影响进展期肺鳞癌患者一线治疗PFS的独立预测因子。

### 2.7 肺鳞癌患者免疫治疗相关不良反应发生情况（表 3）

**表 3:** 免疫治疗相关不良反应发生情况 Incidence of immunotherapy-related adverse events

Adverse events	Grade 1			Grade 2			Grade 3			Grade 4	
	Elderly	Younger		Elderly	Younger		Elderly	Younger		Elderly	Younger
Pneumonia	1	1		5	5		1	6		0	2
Myocarditis	0	0		1	1		0	0		0	0
Liver injury	0	1		0	0		0	0		0	0
Rashes	4	4		3	6		4	2		0	0
Hypothyroidism	0	1		1	0		1	1		0	0
Nephropathy	0	0		0	1		0	1		0	0
Arthritis	1	0		0	0		1	0		0	0
Cholecystitis	0	0		0	1		0	0		0	0
Enteritis	1	0		0	0		0	0		0	0
Feeble	0	3		1	4		2	3		0	0
Anorexia	0	1		2	3		1	2		0	0
Peripheral neuropathy	1	0		0	1		0	0		0	0

185例患者中，93例疗程中曾接受过免疫单药治疗或与化疗联合，可统计免疫相关不良反应的88例患者中，总体不良反应发生率为58.0%（*n*=51），其中3级及以上不良反应发生率为25.0%（*n*=22）。高龄组与低龄组总体不良反应发生率与3级及以上不良反应发生率均无差异，分别为64.7% *vs* 53.7%，23.5% *vs* 25.9%（χ^2^=0.034和χ^2^=0.06，*P*均 > 0.05）。低龄组中2例患者出现4级免疫治疗相关性肺炎。最常见的3级及以上不良反应包括：皮疹6例（11.8%），免疫相关性肺炎9例（17.6%），乏力5例（9.8%），甲状腺功能减退2例（3.9%），免疫相关性关节炎1例（2.0%），免疫相关性肾病1例（2.0%），均在激素及对症治疗后好转。

## 3 讨论

大多数肺鳞癌患者有重度吸烟史，导致了基因组的高度不稳定性和整体突变负荷增高^[6, 16]^。基因组不稳定性可引起新抗原的产生，从而为肺鳞癌患者从以ICIs为代表的免疫治疗中获益提供了依据。近年来，ICIs的出现改变了肺鳞癌的治疗模式^[8, 17, 18]^。Lee等^[19]^发现抗PD-1/PD-L1治疗可以为肺鳞癌患者带来显著生存获益和提高客观缓解率。此外，一些免疫单药治疗或免疫联合化疗研究，如RATIONALE-307^[17]^、KEYNOTE-407^[8]^和CheckMate-017^[6]^表明，ICIs提高了晚期肺鳞癌的生存率，并为一线/二线治疗提供了新的选择。

在免疫单药作为一线方案治疗晚期肺鳞癌的探索中，KEYNOTE-024研究纳入PD-L1高表达（≥50%）的晚期NSCLC患者，对比Pembrolizumab单药与化疗的疗效，Pembrolizumab组较含铂双药化疗组显著提高OS（44.8% *vs* 27.8%），提高了鳞癌亚组患者的mPFS^[20]^。KEYNOTE-042研究^[8]^中，纳入PD-L1表达≥1%的初治晚期NSCLC患者^[3]^，在TPS≥1%的鳞癌亚组中死亡风险降低了25%（HR=0.75）。KEYNOTE-407研究对比免疫联合化疗与单纯化疗的疗效。紫杉醇/白蛋白紫杉醇+卡铂联合Pembrolizumab较单纯化疗明显延长OS和PFS，分别为15.9个月 *vs* 11.3个月、6.4个月 *vs* 4.8个月。ORIENT-12研究比较国产PD-1抑制剂信迪利单抗+GP方案与单纯化疗的疗效，联合组降低复发风险46.4%（HR=0.536）^[21]^。CameL-sq研究^[22]^对比卡瑞丽珠单抗联合紫杉醇+卡铂与化疗，联合治疗组显著延长PFS和OS，分别为8.5个月 *vs* 4.9个月，未达到 *vs* 14.5个月。

本研究中无论高龄组或低龄组，联合治疗组ORR均明显优于化疗组，分别达到69.2%和53.8%，与Wang等^[17]^和Ren等^[22]^的研究中联合治疗组ORR（分别为72.5%和64.8%）相接近。高龄组与低龄组ORR无明显差异。两个年龄组中，联合组mPFS和mOS均较化疗组明显延长，尤其低龄组获益更加明显，与KEYNOTE-407研究和CameL-sq研究结果相类似。高龄组中，免疫单药mPFS较化疗组延长，但总生存无获益。低龄组中，免疫单药mPFS和mOS均差于化疗组，与Carbone等^[9]^的研究结果一致。提示无论高龄组或低龄组，免疫单药治疗获益有限，联合治疗优于化疗，可作为体能状况良好进展期肺鳞癌患者一线治疗优先选择。考虑免疫单药组入组人数有限，需进一步验证。

目前缺乏老年肺鳞癌患者免疫治疗相关临床研究。有研究^[13]^表明老年肺鳞癌患者可获得与中青年患者相似的有效率和毒性反应发生率。一项聚类研究^[14]^汇总分析3项试验中≥75岁患者的数据，PD-L1阳性晚期NSCLC患者中，Pembrolizumab较化疗改善了OS，增龄与Pembrolizumab治疗的毒性增加无关。

我们的研究中，无论免疫单药、免疫联合化疗，高龄组患者的ORR、mPFS和mOS均与低龄组无差异。总体不良反应发生率与3级以上不良反应发生率两组相类似。3级以上不良反应主要表现为皮疹、免疫相关性肺炎和乏力等，与既往研究结果^[20]^一致，且高龄组未发生4级及以上或不可治疗的不良反应。提示老年肺鳞癌患者一线接受免疫联合化疗可获得与其他年龄组相似的安全性、有效率和生存获益。

2020年*Immunity*杂志提出的免疫治疗十大挑战包括通过综合生物标志物来实现最大化的个体治疗突破。2021年美国临床肿瘤学会（American Society of Clinical Oncology, ASCO）发布的肿瘤进展年度报告更是确定策略，要更好地预测患者对免疫疗法的反应和耐药、识别与免疫治疗相关的血液和组织生物标志物。我们分析高龄组与低龄组PD-L1和TMB表达情况及与预后的关系。高龄组PD-L1表达阳性（≥1%）率显著高于低龄组（82.6% *vs* 54.3%），PD-L1表达50%以上阳性率也高于低龄组。两组TMB表达无差异。

高龄组PD-L1表达与生存无关，低龄组中PD-L1阴性患者表现为mOS获益，但未达到统计学差异。PD-L1表达评价受检测抗体不同、评分系统不一致及阈值不同等影响^[23]^，作为ICIs疗效和预后标志物存在一定局限性。高TMB表达患者中，联合治疗组mPFS明显优于化疗组。低TMB患者中，联合组mPFS低于化疗组。提示TMB表达是肺鳞癌患者一线免疫治疗疗效预测因子，与CheckMate 227研究^[24]^相一致。高龄组TMB-H较TMB-L患者延长mOS，但低龄组中TMB-H与TMB-L患者mOS无差异。多因素分析TMB不是独立预后因子。总之，PD-L1和TMB作为进展期肺鳞癌患者免疫治疗疗效预测和预后标记，用于筛选ICIs受益人群方面有待进一步证实。开发更有效的生物标志物对指导肺鳞癌免疫治疗具有重要意义。本研究中TMB-L患者中，化疗组mPFS明显延长，考虑与部分患者合并放疗及病例数较少有关，人群选择不同可对结果产生一定影响。

高龄组肺鳞癌患者免疫治疗的ORR、mPFS和mOS与低龄组相似。免疫联合化疗较化疗获得更高的疾病缓解率、更长的PFS和OS。免疫单药较化疗无明显生存获益。高龄组患者对免疫单药或联合化疗总体不良反应可耐受，与低龄组无明显差异。PD-L1和TMB作为进展期肺鳞癌患者免疫治疗疗效和预后因子的价值有待进一步评价。本研究为回顾性研究，受纳入样本量、治疗药物不同等影响，相关结果仍需大样本或随机对照研究进一步证实。
